# Latinx individuals’ knowledge of, preferences for, and experiences with prenatal genetic testing: a scoping review

**DOI:** 10.1186/s12978-022-01438-2

**Published:** 2022-06-06

**Authors:** Natalie Grafft, Andrew A. Dwyer, María Pineros-Leano

**Affiliations:** 1 School of Social Work, Boston College, Chestnut Hill, MA 20467 USA; 2 William F. Connell School of Nursing, Boston College, Chestnut Hill, MA 02467 USA; 3 Massachusetts General Hospital-Harvard Center for Reproductive Medicine, Massachusetts General Hospital, Boston, MA 02115 USA

**Keywords:** Health literacy, Genetic counseling, Genetic testing, Genomic healthcare, Genetic literacy, Attitudes, Prenatal testing, Latinx populations

## Abstract

**Background:**

The American College of Obstetricians and Gynecologists recommends prenatal genetic testing (PGT) be offered to all pregnant persons regardless of known risk factors. However, significant racial/ethnic differences exist regarding acceptance of PGT contributing to disparities. Latinas (Latinx), one of the fastest growing ethnic groups in the United States, have low PGT acceptance rates. This systematic scoping review aimed to provide a landscape of existing literature on Latinx individuals’ knowledge of, preferences for, and experiences with prenatal and preconception genetic testing. Synthesizing the current state of the science may inform development of culturally tailored interventions to support high-quality PGT decisions (e.g., informed, aligned with a pregnant persons’ values).

**Methods:**

We conducted a structured, systematic literature search of published articles and gray literature in electronic databases (PubMed, PsycINFO, CINAHL, Medline, Embase, Eric, Social Services Abstracts, and PsycArticles). Articles in English published prior to March 2021 were retrieved relating to genetics, pregnancy, and Latina women. Articles underwent title, abstract and full-text review by independent investigators to assess inclusion and exclusion criteria. Risk of bias was evaluated by two investigators. Iterative thematic analysis was employed to group study findings into themes to identify possible targets for interventions.

**Results:**

The search generated 5511 unique articles. After title screening, 335 underwent abstract review and subsequently 61 full-text review. Twenty-eight studies met inclusion criteria and 7 additional studies were included after reviewing reference lists. Three overarching themes emerged: genetic knowledge/literacy (26/35, 74%), provider (mis)communication/patient satisfaction (21/35, 60%), and cross-cultural beliefs (12/35, 34%). Studies indicate discordant patient-provider language (n = 5), miscommunication (n = 4), and lack of concordant decision-making (n = 4) pose barriers to high-quality PGT decisions. Immigration status (n = 1) and religious beliefs (n = 5) are additional factors influencing PGT decisions.

**Conclusions:**

Identified studies suggest that cultural and linguistic factors affect Latinx PGT decision-making. Latinx individual’s comprehension and recall of PGT information is enhanced by culturally and linguistically concordant providers—suggesting that culturally-informed interventions may enhance PGT acceptability and support high-quality decisions. Future directions to surmount PGT disparities may include community health workers and cultural brokers to empower Latinx people to make informed decisions aligned with their values and preferences.

**Supplementary Information:**

The online version contains supplementary material available at 10.1186/s12978-022-01438-2.

## Plain language summary

Significant racial, ethnic, and language disparities exist in prenatal genetic testing (PGT). Latina (Latinx) people, one of the fastest growing ethnic groups in the United States, have low acceptance rates of PGT. This scoping review provides a systematic search of the literature to better understand Latinx individuals’ knowledge of, preferences for, and experiences with PGT. Eight electronic data bases were systematically searched and identified articles underwent title, abstract, full text, and reference review. Iterative thematic analysis was conducted to group article findings into themes. Thirty-five studies met inclusion criteria and three overarching themes were identified: genetic knowledge/literacy, provider (mis)communication/patient satisfaction, and cross-cultural beliefs. Findings indicate that discordant patient-provider decision making and language and patient provider miscommunication pose barriers to high-quality PGT decisions. Latinx individuals’ understanding and recall of PGT information is improved when delivered in a culturally and linguistically concordant manner. This suggests culturally-informed interventions, including the use of community health workers or cultural brokers, may enhance PGT acceptability and support high quality pregnancy decisions.

**Supplementary Information:**

The online version contains supplementary material available at 10.1186/s12978-022-01438-2.

## Background

Significant technologic advances including next-generation sequencing technologies and novel bioinformatic pipelines have advanced the use of genomic information in healthcare. In obstetrics, prenatal genetic testing (PGT) is used to assess a person’s risk of carrying a fetus with a chromosomal disorder. The American College of Obstetricians and Gynecologists (ACOG) recommends PGT should be offered to all pregnant people regardless of known risk factors [1]. There are a range of PGT options to detect chromosomal aneuploidies (e.g., too few or too many chromosomes) and choices can be complex—as each test has respective advantages and limitations. Screening test options include the first trimester (10–13 weeks), quad screen (15–22 weeks) and more recently, noninvasive prenatal testing (NIPT) using cell-free DNA (cfDNA) [2]. Genetic screening results can help reassure individuals of a low likelihood of a fetal abnormality or inform the obstetrician and patient of a possible genetic condition warranting altered management plans. A positive screening test result triggers subsequent discussion of additional diagnostic testing options such as amniocentesis, chorionic villus sampling (CVS), and fetal chromosomal microarray testing [3]. Importantly, ACOG advocates that pregnant individuals be clearly informed that both screening and diagnostic tests are optional (e.g., not mandatory) and shared decision-making is a critical component of testing decision-making [1].

Pre-test genetic counseling is an important part of supporting high-quality genetic testing decisions (e.g., informed and aligned with the patient’s values and preferences). Genetic counseling combines patient education and non-directive counseling techniques to educate patients about potential risks/benefits, possible test results and their implications, as well as limitations of genetic testing [4]. Thus, genetic counselors aim to provide clear information, elicit values/beliefs and invite reflection to support high-quality decisions for genetic testing. While genetic testing technologies are increasingly integrated into care pathways, advances in genomic healthcare have not benefitted all populations equally. A 2018 report from the National Academies of Sciences, Engineering and Medicine notes growing disparities in genomic healthcare [5]. Notably, significant racial/ethnic and language differences exist regarding acceptance of genetic testing [6]. Individuals from racial and ethnic minority groups are less likely than non-Hispanic White people to have PGT [7]. Evidence indicates Latinx individuals have significantly lower acceptance rates of prenatal diagnostic testing than their White and Black peers [8]. Also, data show Spanish-speakers are less likely to recall prenatal diagnostic testing discussions with their healthcare provider [9]—raising important ethical concerns regarding the informed consent process. Further, Latinx individuals are less likely to have a preference concordant decision-making process (e.g., aligned with preference for autonomous, shared, or provider-driven decision making respectively) [10]. These data point to significant PGT disparities for Latinx individuals. Such disparities are highly relevant as the Latinx population represents 18% of the United States (U.S.) population and accounts for 28% of children under 18-years of age [11].

We conducted a scoping review to provide a comprehensive review of the literature using qualitative, quantitative, and mixed-methods to chart the current understanding of Latinx people’s knowledge, values, preferences, and experiences with PGT. Providing a landscape of the current state of the science is a rational step for understanding the structural (e.g., health system, healthcare providers) and human factors (e.g., literacy/numeracy, attitudes, knowledge, beliefs) affecting uptake of PGT among Latinx individuals. We aim to synthesize the existing literature to guide the development of culturally-informed interventions to support Latinx individuals in making high quality pregnancy decisions and to reduce genomic health disparities.

## Methods

We conducted a comprehensive, systematic scoping review to chart the current understanding of Latinx people’s knowledge, values, preferences and experiences with PGT. A scoping review was conducted, over a systematic review, due to the broad nature of our research question and desire to summarize and disseminate findings in order to inform future research and interventions [12]. We employed the five-stage Arksey and O’Malley framework for scoping reviews [12] which overlap with the sequential steps of a systematic review.

### Identifying the research question

This scoping review was guided by two inter-related questions: What is the understanding of, preferences for, and experiences with preconception and prenatal genetic testing and counseling among Latinx pregnant people living in the U.S.? What is known about prenatal genetic literacy and numeracy in Latinx people living in the U.S.?

### Identifying relevant literature

We used a two-tiered approach to identify relevant articles. First, we conducted a structured, systematic search in 8 electronic data bases (PubMed, PsycINFO, CINAHL, Medline, Embase, Eric, Social Services Abstracts, and PsycArticles) using search terms related to genetics (Genet*, genetic literacy, genetic counseling, genetic education, heredit*, inherit*), pregnancy (prenatal*, pre-natal, perinatal*, antenatal*, ante natal*, preconception*, pre conception*, family NEAR/3 plan*, pregna*), and Latina women (latin*, hispan*, latin American, cuba*, mexic*, salvador*, guatemal*, nicarag*). All articles were exported into Endnote ™ and duplicates were removed. Second, we employed a “snowball” technique to identify additional articles not found in the structured search. The “snowball” method involved reviewing the reference lists of included articles to identify additional relevant studies.

### Selecting the literature

Articles included in this scoping review met specific inclusion criteria: (1) primary research studies, (2) systematic reviews and meta-analyses, (3) studies concerning Latinx individuals living in the U.S. who received preconception/ prenatal genetic testing/ counseling and (4) studies with at least 40% of the sample identifying as Latinx (or studies that differentiated results by race/ethnicity). Case reports, opinion pieces, review articles, studies regarding in vitro fertilization, and studies on Latina women under the age of 18 years were excluded from the review.

**Fig. 1:**
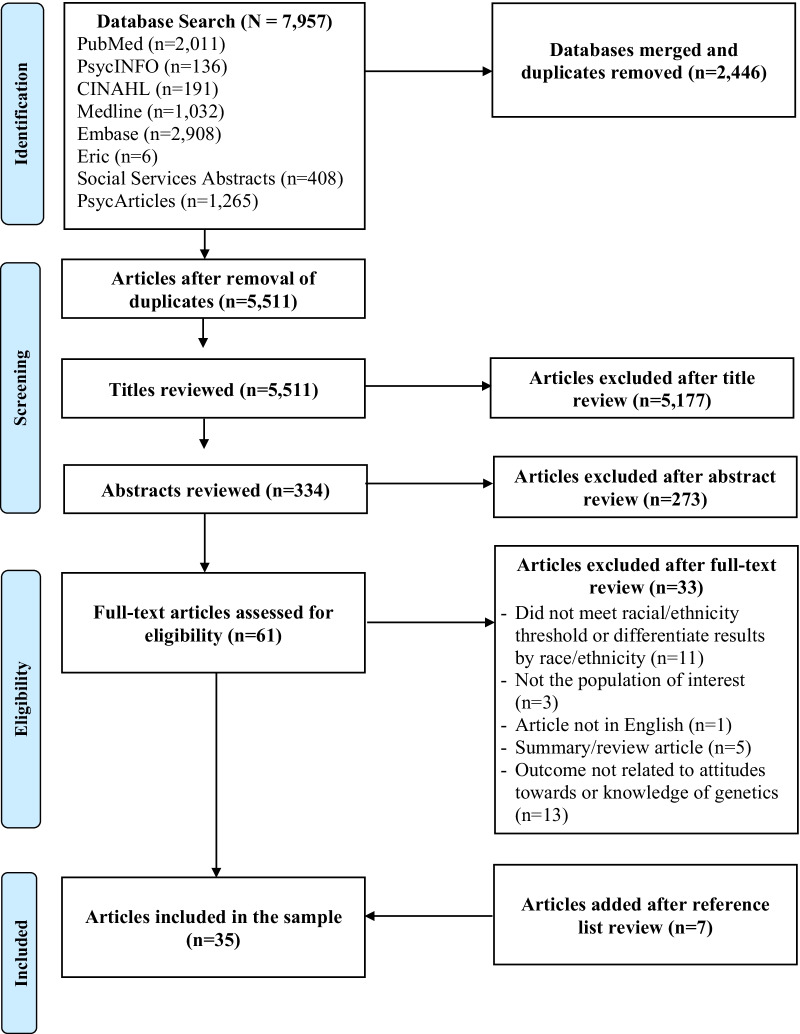
Flow diagram of search results

The database search yielded 5511 articles after duplicates (n = 2446) were removed. One researcher (N.G.) reviewed all titles to identify 334 potentially relevant articles. Next, each abstract was independently reviewed by two investigators per the inclusion/exclusion criteria using Rayyan. One author (NG) reviewed all abstracts and two authors (AAD and MPL) each reviewed half of the abstracts. Discrepancies were discussed with the entire research team and resolved by discussion. In total, 61 articles were identified for full-text review. Similar to the abstract review, each article was read in full by two independent investigators (NG reviewed all articles, AAD and MPL each reviewed half of the articles) and a determination to include/exclude was made based on eligibility criteria. After discrepancies were discussed, 28 articles were included for analysis and data extraction. For the second tier (“snowball”) approach, one investigator (NG) examined the reference lists of the 28 studies identifying 7 additional studies meeting inclusion criteria. A total of 35 studies were included in this scoping review (Fig. 1).

### Charting the data

Information pertaining to the study topic, Latinx sample size, data collection methods, research design, validated measurement tools, PGT type, and findings were extracted for each study (N.G.). To assess the methodological rigor of each quantitative study, we used the 8-item JBI Critical Checklist for Analytical Cross-Sectional Studies [13] and 12-item JBI Critical Appraisal Checklist for Randomized Control Trials [14]. Two members of the research team independently scored articles using the checklist to assess risk of bias (NG reviewed all articles, AAD and MPL each reviewed half of the articles). Initial inter-rater agreement for risk of bias assessment was 70.2% and discrepancies were discussed until agreement was reached. Information on the quality of each study obtained from the risk of bias assessment is provided in the Additional file 1.

### Collating, summarizing, and reporting results

Extracted data were summarized in tabular format according to study methodology (e.g., quantitative, qualitative, mixed-methods). Data were then synthesized across the 35 studies using an iterative thematic analysis process [15]. In brief, main study findings were clustered into groups (themes) relating to a shared/similar construct. All investigators discussed the themes in iterative meetings to refine and collapse the groupings into the most salient and coherent themes. The final themes were summarized in tables relating to the respective theme and separated by study methodology. Findings within each theme were used to guide discussion regarding targets for interventions among Latinx individuals to promote high-quality PGT decisions.

## Results

A total of 35 studies were included comprising qualitative (n = 13), mixed-methods (n = 11), and quantitative (n = 11), studies. Risk of bias assessment for the quantitative studies showed mixed results. Studies published before 2015 had moderate to high risk of bias whereas more recent studies (i.e., 2015–present) generally had low risk of bias. In particular, the majority of studies published prior to 2015 neither used objective measurement criteria nor measured outcomes in a valid/reliable manner. Studies with high risk of bias were not excluded from our synthesis of findings and development of three themes. Rather, the risk of bias assessment was taken into account when discussing future directions for research and intervention development. For example, findings from studies with high risk of bias were not given as much weight when considering future directions of research and intervention development.

In total, 34/35 (97%) studies interviewed Latina women, 17 (50%) of which focused exclusively on the Latino population. Eight (23%) studies observed genetic counseling or educational sessions, five (14%) studies included male partners, three (9%) studies interviewed providers, and two (6%) studies performed chart reviews. The vast majority (19/28, 68%) of studies did not indicate the language in which participants received genetic information. Two (6%) studies specified that genetic information was received in Spanish, two (6%) studies indicated information was communicated via medical interpreters, one (3%) study indicated information was provided by a bilingual provider/interpreter and two (6%) studies indicated information was provided in the patient’s preferred language. Broadly, results of the identified studies spanned three themes: genetic knowledge/literacy, provider (mis)communication/patient satisfaction, and cross-cultural beliefs.

### Genetic knowledge/literacy

Twenty six of 35 (74%) studies reported on genetic literacy in Latina women (Table 1). Genetic literacy can be defined as “sufficient knowledge and understanding of genetic principles to make decisions that sustain personal well-being and effective participation in social decisions on genetic issues” [16]. Few studies have used a validated measure to assess genetic knowledge or literacy in Latina women. Two (8%) studies used the Rapid Estimate of Adult Literacy in Medicine-Revised Scale (REALM-R) [9, 10], one (4%) the Lipkus Expanded Numeracy Scale [10] and another (4%) used the Rapid Estimate of Adult Literacy in Genetics (REAL-G) [17] to assess genetic literacy and numeracy. Overall, the majority of studies found that even after receiving educational interventions or genetic counseling, Latina women did not have optimal knowledge of PGT [17–25]. Two studies utilizing linguistically concordant providers to communicate genetic information found Latina women were able to better understand and retain complex genetic information [26, 27].

**Table 1:** Characteristics of studies with the theme ‘genetic knowledge/literacy’

Author (year)	Data collection method (n = sample size; all sample sizes are restricted to Latina patients except the provider sample) [genetic testing type]	Nativity	Country of origin	Findings [validated measurement tool]
*Qualitative*				
Press (1993) [23]	Patient interview (n = 20); provider interview (n = not disclosed); observation (n = 20) [AFP]	Not assessed	Mexico	(1) Seventy five percent of the participants said they read and understood the written AFP information, but based on knowledge questions very little information was retained
Browner (1995) [25]	Patient interview (n = 20); observation (n = 35) [AFP]	Not assessed	Mexico	[Marin Acculturation Scale] (1) Participants retained very little information on PGT after intake appointments with providers and supplemental educational pamphlets
Freda (1998) [20]	Patient interview (n = 31) [AFP]	Not assessed	Not assessed	(1) After viewing an informational video on AFP, 38% of the participants did not know the purpose of AFP, 72% of the participants believed a negative AFP test meant a healthy fetus, many knowledge questions had an 80% incorrect answer rate, and 45% of participants understood the recommended medical follow up for a positive test
Griffiths (2008) [22]	Patient interview(n = 33) [AFP, Amnio]	Foreign born and US born	Mexico	[Marin Short Acculturation Scale] (1) One third of participants thought there had to be a reason for birth defects and 1/3 of participants thought birth defects could be random. (2) One third of participants thought AFP results were diagnostic and were unaware a positive test result would result in more screenings. (3) Most felt that a negative AFP result guaranteed a healthy baby. (4) Participants reported they would decline amniocentesis due to risks associated with the procedure
Markens (2010) [38]	Patient interview (n = 147) [AFP, Amnio]	Foreign born and US born	Mexico	(1) Participants who declined amniocentesis were more skeptical of scientific knowledge and mistrusted medical institutions than participants who accepted amniocentesis
Thompson (2015) [33]	Patient interview (n = 25) [General PGT]	Foreign born	El Salvador, Honduras, Mexico	(1) Participants declined testing due to risks associated with the procedures
Floyd (2016) [21]	Patient interview (n = 10) [cfDNA]	Not assessed	Not assessed	(1) After genetic counseling, participants had difficulty distinguishing the different PGT options. (2) Spanish-speaking participants chose to undergo cfDNA because of their doctor’s recommendation
Garza (2019) [27]	Patient interview (n = 20) [General PGT]	Foreign born	Cuba, El Salvador, Guatemala, Honduras, Mexico	(1) After genetic counseling sessions with a Spanish-speaking genetic counselor, participants were able to recall genetic information, including vocabulary terms. (2) Participants consulted close family/friends to help support them through making the decision of whether to undergo PGT. (3) Participants were receptive to the medical information and trusted their healthcare providers. (4) Participants chose to undergo NIPT rather than amniocentesis due to risks associated with amniocentesis
Page (2021) [17]	Patient interview (n = 10) [General PGT]	US born	Not assessed	[REAL-G Genetic Literacy Scale] (1) Ninety percent of participants first learned of PGT through their healthcare provider and 70% had no previous knowledge of genetic abnormality terms or PGT options
*Quantitative*				
Browner (1996) [18]	Patient interview (n = 56) [AFP]	Not assessed	Mexico	(1) No difference in AFP knowledge scores for participants who accepted or declined PGT. (2) Participants who received video education on AFP, in lieu of written information, retained more AFP knowledge 3 months later. (3) Education had the strongest effect on AFP knowledge and Mexican–American and European-American participants had no statistically significant difference in AFP knowledge scores. (4) Mexican immigrant participants had significantly lower AFP knowledge scores than Mexican–American and European-American participants
Learman (2003) [40]	Patient interview (n = 220) [General PGT]	Foreign born & US born	Not assessed	(1) Medical providers had a larger influence on participants' PGT decisions than friends or religious leaders had
Singer (2004) [39]	Men/women of childbearing age interview (n = 428) [General PGT]	Not assessed	Not assessed	(1) Latino and Black participants had a higher preference for PGT than White participants. (2) Latino participants were similar to White participants in their knowledge of PGT, interest in the topic, and confidence in medical institutions. (3) One third (32.3%) of the Latino participants who did not read scientific news sited their doctor as the most important source of knowledge
Case (2007) [36]	Women of childbearing age interview (n = 284) [General PGT]	Not assessed	Not assessed	(1) Seventy eight percent of participants stated they would undergo PGT with Black and Latina participants more likely to want PGT than their White participants. (2) Ninety five percent of participants wanted to undergo testing to receive treatment in utero
Hawk (2011) [19]	Patient interview (n = 48) [General PGT]	Foreign born & US born	Not assessed	(1) Knowledge of genetic conditions did not vary in participants who chose to undergo PGT versus those who chose not to undergo testing. (2) After prenatal genetic counseling sessions, knowledge of abnormalities was deficient for 50% of participants. (3) There were no differences in English and Spanish-speaking participants for reasons to undergo (or not to undergo) PGT. Reasons to undergo PGT were that their doctor recommended it and to be reassured their pregnancy was normal. Reasons not to pursue PGT was that the participant would never consider terminating the pregnancy
Kupperman (2014) [30]	Patient interview (n = 322) [General PGT]	Not assessed	Not assessed	[Decisional Conflict Scale & Decision Regret Scale] (1) Differences in prenatal knowledge and decisional regret were examined between participants who were randomized to an intervention group, received prenatal genetic education and decisional support, and participants who received care as usual. Participants in the intervention group had higher knowledge scores and were more likely not to undergo PGT. There were no differences in decisional regret between participants in the intervention and control groups
Bryant (2015) [9]	Patient interview (n = 322) [General PGT]	Not assessed	Not assessed	[Rapid Estimate of Adult Literacy in Medicine-Revised] (1) Utilized a validated measurement tool to assess for genetic literacy
Molina (2019) [10]	Patient interview (n = 292) [General PGT]	Not assessed	Not assessed	[REALM-R & Lipkus Expanded Numeracy Scale] (1) Study used a validated scale to assess for genetic knowledge. (2) Latina participants (both Spanish and English speaking) had lower health numeracy than White participants
*Mixed methods*				
Mittman (1998) [26]	Patient interview (n = 826); observation (n = not disclosed) [Amnio; CVS]	Foreign born & US born	Not assessed	(1) Eighty one percent of participants had an increase in genetic knowledge after a genetic counseling session facilitated by a genetic counselor and cultural broker. (2) There was no correlation between genetic knowledge and acceptance or refusal of amniocentesis
Penchaszadeh (1998) [35]	Patient interview (n = 100) [Amnio]	Not assessed	Not assessed	(1) Fear of risks and pain from the procedure were common reasons participants declined amniocentesis. (2) Fifty three percent of participants believed a normal amniocentesis test result indicated the fetus was healthy in all domains. (3) Thirty one percent of participants' PGT decisions were influenced by their provider
Press (1998) [28]	Patient interview (n = 75); chart review (n = 298) [AFP]	Foreign born & US born	Mexico	[Marin Short Acculturation Scale] (1) Spanish speaking Latina participants were almost 2 × as likely (1.94 odds ratio) to refuse AFP testing than pregnant participants (of varying backgrounds) in the sample. (2) Accepting or refusing genetic testing was not related to AFP knowledge. (3) Spanish speaking Latina participants refused AFP testing out of fear it would lead to amniocentesis; amniocentesis was feared due to the procedure itself and associated risks
Browner (1999b) [32]	Patient interview (n = 147); chart review (n = 379) [Amnio]	Foreign born and US born	Mexico	(1) Participants accepted amniocentesis to maintain a good relationship with their medical team. (2) The participants’ understanding of the risks of the procedure, fear of birth defects, and rapport with the medical team were influential factors of whether to undergo amniocentesis. (3) Fear of the procedure was a reason participants refused amniocentesis. (4) Participants who were skeptical of medicine and their doctor’s recommendations were more likely to decline amniocentesis
Browner (2000b) [37]	Patient interview (n = 147) [AFP, Amnio]	Foreign born and US born	Mexico	[Marin Short Acculturation Scale] (1) Participants who were skeptical of medicine and science were more likely to decline PGT than participants with positive attitudes towards science. (2) Participants who trusted their doctor were more likely to accept PGT than participants who did not value their doctor’s recommendations
Browner (2000a) [34]	Patient interview (n = 43) [Amnio]	Foreign born	Mexico	[Marin Short Acculturation Scale] (1) Seventy one percent of the participants who refused and 46% of participants who accepted amniocentesis thought the procedure was extremely risky for the fetus. (2) Participants had several misconceptions about the amniocentesis procedure. (3) Participants agreed to undergo amniocentesis in order to be viewed positively by their providers
Browner (2003) [41]	Patient interview (n = 120); observations (n = 77) [Amnio]	Foreign born and US born	Mexico	(1) Genetic counselors were not a trusted source of knowledge for participants with limited educational background
Hunt (2005) [24]	Patient interview (n = 40); provider interview (n = 50); observation (n = 101) [AFP, Amnio]	Foreign born and US born	Mexico	(1) Seventy eight percent of participants stated they took into consideration their doctor’s recommendation when deciding whether to undergo amniocentesis. (2) There were statistically significant differences in the amount of information provided by certified genetic counselors and non-certified genetic counselors. Participants, however, had no statistically significant difference in recollection of information
Farrell (2015) [29]	Patient interview (n = 63) [NIPT]	Foreign born and US born	Not assessed	(1) Thirty five (34.9%) percent of participants underwent NIPT; language and education were predictive factors. (2) Almost half (47.6%) of participants said they made their testing decisions based on God's will. (3) Participants did not understand the limitations of testing. (4) Knowledge of testing was lower for participants who declined the PGT than for participants who accepted testing

*AFP*  positive alpha-fetoprotein, *CVS*  chorionic villus sampling, *Amnio*  amniocentesis, *NIPT*  non-invasive prenatal testing

Although genetic knowledge was limited, 4/6 (67%) studies did not identify differences in genetic knowledge between Latina women who accepted or declined PGT [18, 19, 26, 28]. Two (2/6, 33%) studies found differences. One study indicated that genetic knowledge was lower in Latinas who declined PGT [29] whereas Kupperman and colleagues (2014) found genetic knowledge to be higher [30] for Latinas who declined testing. Kupperman and colleagues’ (2014) randomized control trial is noteworthy because the intervention focused both on prenatal genetic education and supporting high-quality decisions [30]. Women randomized to the intervention had higher prenatal genetic knowledge scores than women in the control group. However, no between-group differences were observed in terms of decisional regret after accepting or declining testing [30].

In relation to amniocentesis, perceived risks and fear of the testing were common reasons why women declined testing [22, 27, 28, 31–35]. Several studies note Latina women had misconceptions about testing and were unaware of testing limitations [29, 34, 36]. For example, Case and colleagues (2007) found, 640/676 (95%) women opted for PGT to receive in utero medical treatment if an anomaly was detected [36]. Further, many Latina women considered a negative prenatal genetic test as guaranteeing a healthy fetus and baby [20, 22, 35, 37].

Studies examining differences in Latina women who accepted and refused PGT found differing attitudes towards Western medicine and science (e.g., skeptical of medicine) between women who accepted and declined PGT [32, 37, 38]. Several studies identified that Latina women trusted their health care providers and valued their physicians as key sources of information [21, 27, 35, 39, 40]. However, others note the importance of religious beliefs [38] and familial influences/norms [27] as important mediating factors in PGT decision-making. Browner and colleagues (1999b; 2000a) found that many Latina women in their sample were motivated to accept PGT to maintain a good relationship with their physician [24, 32]—suggesting that hierarchy and power structures influenced decision-making. In contrast, Browner and colleagues (2003) found that using interpreters and ethnically non-concordant providers posed barriers for establishing trust with genetic counselors [41].

### Provider (mis)communication/patient satisfaction

Twenty one of 35 (60%) studies reported findings related to provider (mis)communication and patient satisfaction (Table 2). Notably, miscommunication did not appear to diminish Latina women’s perceived acceptability of genetic counseling [32]. Overall, studies revealed relatively low levels of interaction between Latina women and providers when discussing genetic information [31, 42]. The literature identifies several factors contributing to limited engagement with genetic healthcare professionals including use of interpreters [41, 42], medical jargon [41], and Latina women opting to converse in English (in lieu of Spanish or utilizing an interpreter) [9]. Additionally, four (19%) studies found information provided to Latina women often focused on the test/procedures and related risk factors, rather than presenting information on potential findings (e.g., genetic abnormalities) [23–25, 42, 43]. For example, one study found that in 19/35 (54%) prenatal intakes, participants were told the purpose of PGT (i.e., what the test screened for) whereas 31/35 (89%) prenatal intakes, discussed the timeframe when testing would take place [25]. Further, one study observed genetic counseling sessions and found that Latina patients were not able to engage in conversations about risk (e.g., risk of complications from genetic testing or risk of a fetal anomaly) [43]. Patient demographics do not appear to influence the content delivered in genetic counselling sessions, rather, provider time constraints determined the breadth of information provided and quality of the discussion [25]. Hunt and colleagues (2005) found 25/40 (63%) Latina patients neither understood the reason for PGT nor the reason for being referred to a genetic specialist (following an abnormal screening test result) [24]. Cultural mismatch also appeared to contribute to miscommunication. For example, genetic counseling uses a non-directive approach yet many Latina women prefer a provider-driven approach [10, 26, 32, 41]. A study observing genetic counseling sessions found clinicians were hesitant to address ethnic ‘myths’ out of a desire to respect the patient’s culture—contributing to information gaps [41]. The lack of clarity in communication during genetic counseling sessions often led Latina women to decline genetic testing and further counseling [17, 41]. Another source of miscommunication relates to discussions of the financial aspects of PGT. Discussion of financial considerations for PGT are not universally discussed in pre-test counseling and one study revealed Latina women desired more information on costs and financial implications of genetic testing [44]. Cumulatively, these various factors affect interactions with genetic counselors and potentially undermine the quality of genetic counseling encounters.

**Table 2:** Characteristics of studies with the theme ‘mis(communication)/patient satisfaction’

Author (year)	Data collection method (n = sample size; all sample sizes are restricted to Latina patients except the provider sample) [genetic testing type]	Nativity	Country of origin	Findings [validated measurement tool]
*Qualitative*				
Press (1993) [23]	Patient interview (n = 20); provider interview (n = not disclosed); observation (n = 20) [AFP]	Not assessed	Mexico	(1) Oral and written AFP related information provided in prenatal intakes was limited with most of the information being focused on the procedure itself. (2) Participants were satisfied with the amount of genetic information received. (3) Participants wanted AFP testing for reassurance that their pregnancy was normal
Browner (1995) [25]	Patient interview (n = 20); observation (n = 35) [AFP]	Not assessed	Mexico	[Marin Acculturation Scale] (1) Content delivered during PGT consultations was determined by how rushed the provider was rather than patient demographics. (2) Participants were satisfied with provider discussions on PGT. (3) Fifty four percent of participants were told what AFP screened for and 89% were told that AFP needed to be completed between 15 and 20 weeks of pregnancy
Markens (2003) [46]	Patient interview (n = 157) [AFP, Amnio]	Foreign born & US born	Mexico	(1) The participants’ male partners were often unable to attend genetic counseling appointments due to work related obligations/financial constraints. (2) Many participant couples expressed joint responsibility for parenting yet said female participants were responsible for deciding whether to undergo amniocentesis
Hunt (2006) [43]	Patient interview (n = 40); provider interview (n = 50); observation (n = 101) [amnio]	Foreign born & US born	Mexico	(1) Provider participants primarily discussed the patients’ risk status (either in regards to complications from the procedure or risk of fetal anomaly) during genetic counseling sessions. (2) Patient participants had difficulty engaging in conversations around risk, as their main concern was reassurance of a healthy pregnancy and fetus
Thompson (2015) [33]	Patient interview (n = 25) [General PGT]	Foreign born	El Salvador, Honduras, Mexico	(1) Participants were satisfied with genetic counseling sessions; every participant stated that all of their questions were answered and felt they had enough information to make an informed decision about PGT. (2) Eight participants said they would prefer group genetic counseling sessions to gain peer support and knowledge. Thirteen participants preferred individual sessions. (3) Participants wanted genetic counseling information in written form so they could take it home after the session
Floyd (2016) [21]	Patient interview (n = 10) [cfDNA]	Not assessed	Not assessed	(1) Spanish-speaking participants wanted genetic information in written form whereas English-speaking participants thought written information was not necessary, as they would turn to the internet for more information. (2) cfDNA was viewed as a way to prepare emotionally for the baby
Garza (2019) [27]	Patient interview (n = 20) [General PGT]	Foreign born	Cuba, El Salvador, Guatemala, Honduras, Mexico	(1) After a genetic counseling session with a Spanish-speaking genetic counselor, participants were satisfied with the amount of information received, valued PGT, liked how genetic counselors empowered them to make their own decisions about PGT, and preferred genetic information to be delivered verbally during genetic counseling sessions rather than in written form. (2) Thirty percent of participants chose to undergo PGT to learn more about their pregnancy
Page (2021) [17]	Patient interview (n = 10) [General PGT]	US born	Not assessed	[REAL-G Genetic Literacy Scale] (1) One hundred percent of participants said providers should describe PGT options and genetic abnormalities in lay terms. (2) Participants stated PGT would better prepare them, emotionally and financially, for their baby. (3) Lack of clarity from providers and risks associated with PGT were reasons participants declined PGT
*Quantitative*				
Hawk (2011) [19]	Patient interview (n = 48) [General PGT]	Foreign born and US born	Not assessed	(1) Eighty five percent of Spanish-speaking participants believed printed information that accompanied prenatal genetic counseling sessions would be helpful in comparison to 47% of English-speaking participants
Bryant (2015) [9]	Patient interview (n = 322) [General PGT]	Not assessed	Not assessed	[Rapid Estimate of Adult Literacy in Medicine-Revised] (1) Latina participants who chose to complete a genetic counseling session in English were less likely to understand that PGT was optional than White participants
Wagner (2018) [44]	Patient interview (n = 70) [General PGT]	Not assessed	Not assessed	[Health Insurance Literacy Measurement Tool (HILMT)] (1) Latinx participants were 2.59 times more likely to want insurance information from their genetic counselors than White participants. (2) Latinx participants were also 2.31 time more likely to expect their genetic counselor to provide exact out of pocket costs than White participants
Ault (2019) [42]	Observation (n = 7) [General PGT]	Foreign born and US born	Not assessed	The researchers analyzed the differences between English-speaking genetic counseling sessions and genetic counseling sessions that were conducted by an English-speaking genetic counselor and a Spanish certified medical interpreter. (1) In comparison to English sessions, there was limited back channeling in the Spanish sessions; this disinvites individuals from expressing themselves. (2) The length of the Spanish and English sessions was similar but the content was not. More words were spoken by both the participant and genetic counselor in the English-speaking sessions than in the Spanish sessions. Genetic counselors spent the same proportion of time talking but said more and provided more clarity in the English sessions. (3) Participants in the English-speaking sessions were more likely to ask questions. (4) Back channeling, used to evaluate engagement, was more common in the English sessions
Molina (2019) [10]	Patient interview (n = 292) [General PGT]	Not assessed	Not assessed	[REALM-R & Lipkus Expanded Numeracy Scale] (1) Spanish-speaking participants preferred a provider driven decision-making process. (2) Compared to White participants, Latina participants were less likely to have a preference-concordant decision process
*Mixed methods*				
Mittman (1998) [26]	Patient interview (n = 826); observation (n = not disclosed) [Amnio; CVS]	Foreign born and US born	Not assessed	(1) The non-directive genetic counseling led participants to seek advice from non-medical experts. 2) Participants understood genetic information when it was delivered in a culturally/linguistically concordant manner
Penchaszadeh (1998) [35]	Patient interview (n = 100) [Amnio]	Not assessed	Not assessed	(1) Reassurance of a normal pregnancy was the reason 31% of the participants wanted to have amniocentesis
Browner (1999a) [31]	Patient interview (n = 129); observation (n = 65); chart review (n = 379) [Amnio]	Foreign born and US born	Mexico	(1) Participants whose partner was present during the prenatal genetic counseling session were more likely to accept amniocentesis, but female participants had more of an influence on the amniocentesis decision than their male partners. (2) Fourteen percent of participants decided whether to undergo amniocentesis or not prior to the genetic counseling session. (3) Low levels of interaction between participants and genetic counselors was observed. (4) Participants were concerned about the pain and risks of amniocentesis. (5) Male partners acted as a liaison between medical providers and female participants
Browner (1999b) [32]	Patient interview (n = 147); chart review (n = 379) [Amnio]	Foreign born and US born	Mexico	(1) Thirty two percent of participants stated they wanted their genetic counselor to be more direct with recommendations about genetic testing. (2) Satisfaction and miscommunication between participants and genetic counselors were high
Moyer (1999) [45]	Patient interview (n = 10) [AFP, CVS, Amnio]	Not assessed	Not assessed	(1) Eighty three percent of participants felt genetic screening was useful, 67% felt a prenatal diagnosis was useful, 83% felt it was important to avoid having a child with a genetic abnormality, and 60% would consider a 1st or 2nd trimester abortion if the fetus was found to have down syndrome
Browner (2000a) [34]	Patient interview (n = 43) [Amnio]	Foreign born	Mexico	(1) Participants accepted amniocentesis for reassurance that their pregnancy was normal
Browner (2003) [41]	Patient interview (n = 120); observation (n = 77) [Amnio]	Foreign born and US born	Mexico	1) The use of medical jargon, inability for genetic counselors to make strong recommendations, and poor translation led to miscommunication between genetic counselors and participants which in turn led participants to decline amniocentesis. (2) The inability for genetic counselors to address 'ethnic myths' out of a desire to respect the patient's culture led to miscommunication and an information gap
Hunt (2005) [24]	Patient interview (n = 40); provider interview (n = 50); observation (n = 101) [AFP, Amnio]	Foreign born and US born	Mexico	(1) Sixty three percent of participants did not understand why they were being referred to a genetic specialist, after an abnormal AFP, nor did they understand the reason for the AFP test. (2) Genetic counselors most often discussed that amniocentesis is optional and the risks of procedure and of anomalies. Less frequently discussed was nature of anomaly and other testing options. (3) Genetic counselors provided too detailed of information which was overwhelming and was not relatable to the participants

*AFP* positive alpha-fetoprotein, *CVS* chorionic villus sampling, *Amnio* amniocentesis, *NIPT*  non-invasive prenatal testing

Several studies document the perceived poor quality of genetic counseling sessions [24, 31, 32, 41, 41]. However, others report Latina women express relatively high levels of satisfaction with genetic counseling [23, 25, 32, 33]. Press and colleagues (1993) found that 30/40 (75%) women reported reading and understanding the educational pamphlets—yet women retained very little information [23]. Several studies support the notion that Latina women value genetic testing [17, 27, 45] and perceive consultations as a means to learn more about their pregnancy [27] as well as plan and prepare emotionally [17, 21, 34, 35, 43] and financially [17] for their baby’s arrival. The literature suggests that Latina women desire information (e.g., risks/benefits of genetic testing procedures, possible results, genetic abnormalities) in lay language to demystify technical medical jargon [17]. Moreover, Latina women appear to value receiving information in written form [19, 21, 33] as it enables more time for comprehension and the ability to share information with family members. This observation is important as male partners may be unable to attend genetic counseling appointments due to work or other constraints [46]. Of note, studies indicate that providing culturally/linguistically concordant genetic counseling improves comprehension [26] and empowers women to make genetic testing decisions—reducing the need for written information [27]. One study examined Latina perspectives on one-on-one versus group counseling models. Approximately one-third (8/25) of participants preferred group sessions whereas more than half (13/25) preferred individual sessions [33]. Group genetic counseling was perceived to be potentially helpful for facilitating exchange of knowledge and resources (e.g., crowdsourcing) as well as peer support. In contrast, confidentiality and individualized recommendations were seen as benefits of individual sessions [33].

### Cross-cultural beliefs

Twelve of 35 (34%) studies reported findings related to cross-cultural beliefs which entails the differing views Latina women and medical providers have on pregnancy (Table 3). Markens and colleagues (2010) noted that Latina women do not experience pregnancy through a medical/scientific lens [38]. Rather, health and illness tend to be viewed through a cultural or metaphysical lens and pregnancy is considered a natural, routine part of a woman’s life [26]—and not a medicalized condition [38]. In comparison to women who identify as Black, Asian, or White, Latina women were more likely to state that in their culture, they learn to accept ‘what is given’ [40]. This cultural perspective is important because it reveals that risk perception may be influenced by personal and cultural experiences as opposed to scientific data [26]. As noted above, faith has been reported to influence “genetic knowledge/literacy”—yet it also affects “cross-cultural beliefs”. While faith does not appear to predict uptake of genetic services [17, 28, 32, 37, 40, 47], many Latina women are guided by faith in their pregnancy-related decision-making [27, 47].

**Table 3:** Characteristics of studies with the theme ‘cross-cultural beliefs’

Author (year)	Data collection method (n = sample size; all sample sizes are restricted to Latina patients except the provider sample) [genetic testing type]	Nativity	Country of origin	Findings [validated measurement tool]
*Qualitative*				
Griffiths (2008) [22]	Patient interview(n = 33) [AFP, Amnio]	Foreign born and US born	Mexico	[Marin Short Acculturation Scale] (1) Birth defects were viewed to be a result of substance use or God’s Will. (2) Diet, low levels of stress, not getting a fright, and engaging in cultural health practices were viewed as ways to avoid birth defects
Markens (2010) [38]	Patient interview (n = 147) [AFP, Amnio]	Foreign born and US born	Mexico	(1) Participants took religion into consideration when deciding whether to undergo genetic testing
Barragan (2011) [48]	Patient interview (n = 15)	Foreign born and US born	Mexico	[Acculturation Rating Scale for Mexican Americans-II] (1) Three of the participants knew someone with down syndrome; two of these participants believed in cultural reasons for down syndrome and specifically believed the abnormality resulted from a strong emotional reaction during pregnancy
Hurst (2011) [49]	Patient interview (n = 11)	Not assessed	Not assessed	(1) Participants believed that traits were passed down one of four ways all being weighted equal; behaviors during pregnancy, genetics, family and community childbearing practices, and God’s will. (2) Behaviors were the primary focus, as they can be controlled whereas genes cannot be altered. (3) Participants comfortably and appropriately intertwined familial and cultural beliefs with medicine to understand genetics and heritability
Seth (2011) [47]	Patient interview (n = 11) [Amnio]	Foreign born	Not assessed	(1) Faith was present in participants who accepted and declined amniocentesis. (2) The perceived risks of the procedure influenced the decision of whether to undergo amniocentesis
Garza (2019) [27]	Patient interview (n = 20) [General PGT]	Foreign born	Cuba, El Salvador, Guatemala, Honduras, Mexico	(1) After the genetic counseling sessions with a Spanish-speaking genetic counselor, participants were able to recall genetic information, including vocabulary terms. (2) Immigration related stressors impact general prenatal care and have unique implications for PGT. (3) Participants’ faith played a role in their PGT decisions
Page (2021) [17]	Patient interview (n = 10) [General PGT]	US born	Not assessed	[REAL-G Genetic Literacy Scale] (1) Participants reported religiosity did not influence their genetic testing decisions
*Quantitative*				
Learman (2003) [40]	Patient interview (n = 220) [General PGT]	Foreign born and US born	Not assessed	(1) Participants' faith did not influence PGT decisions. (2) In comparison to Black, Asian, and White participants, Latina participants were more likely to state that their religious leader would influence their PGT decisions, less likely to be influenced by family members when making PGT decisions, and more likely to state that in their culture they learn to accept what is given
*Mixed methods*				
Mittman (1998) [26]	Patient interview (n = 826); observation (n = not disclosed) [Amnio; CVS]	Foreign born and US born	Not assessed	(1) Participants viewed illness from a cultural lens rather than from a scientific lens. (2) Risk perception of the possibility of a genetic abnormality was influenced by personal experiences and family history rather than scientific data. (3) Linguistically and culturally tailored genetic counseling is useful as participants often made genetic testing decisions based on culture rather than genetic knowledge. (4) A genetic counseling session facilitated by a genetic counselor and a cultural broker led to an increase in genetic knowledge
Press (1998) [28]	Patient interview (n = 75); chart review (n = 298) [AFP]	Foreign born and US born	Mexico	[Marin Short Acculturation Scale] (1) Spanish speaking Latina participants did not view testing as a routine part of prenatal care. (2) Religiously was not associated with genetic testing decisions in Latina participants
Browner (1999b) [32]	Patient interview (n = 147); chart review (n = 379) [Amnio]	Foreign born and US born	Mexico	(3) Religiosity was not predictive of amniocentesis uptake
Browner (2000b) [37]	Patient interview (n = 147) [AFP, Amnio]	Foreign born and US born	Mexico	[Marin Short Acculturation Scale] (1) Participants combined scientific and lay knowledge to understand genetic test results. (2) Religiosity did not predict amniocentesis uptake. (3) Participants thought heredity could be altered through prayer and that the fetus could be altered through non-medical intervention. (4) Participants who declined amniocentesis engaged in an alternate intervention (prayer, reduce physical activity) to help the fetus

*AFP* positive alpha-fetoprotein, *CVS*  chorionic villus sampling, *Amnio*  amniocentesis, *NIPT*  non-invasive prenatal testing

Latina women frequently interpret genetic information (e.g., genetic abnormalities, fetal anomalies) through their individual cultural/religious lens. For example, genetic abnormalities may be thought to result from a strong emotional reaction during pregnancy [22, 48] or God’s will [22]. Evidence suggests that in Latino cultures reducing stress, healthy eating, self-care and engaging in cultural health practices are considered ways to avoid genetic abnormalities [22]. Browner and colleagues (2000b) found that Latina women believe the fetus (and heredity) can be altered through non-medical action (e.g., prayer) [37]. Browner and colleagues (2000b) also noted that women who refused amniocentesis after positive alpha-fetoprotein (AFP) screening almost universally engaged in alternative interventions (e.g., prayer, seeking a traditional healer) [37]. A more recent study found Latina women were able to successfully intertwine traditional cultural beliefs and Western medical knowledge [49]. Specifically, Latina women believed genetics, behaviors during pregnancy, God and community practices contribute equally to pregnancy outcomes. However, Latina women largely focused on behaviors during pregnancy—as they are amenable to change, whereas genetics is non-modifiable [49]. Similar to findings in the other themes, studies suggest that Latina women are better able to understand and recall complex genetic information when providers appropriately incorporate culture into PGT and counseling discussions [26, 27].

It is worthwhile to note that immigration status is distinct from culture. However, it is an important consideration when understanding Latina women’s experiences with pregnancy and interactions with the medical system. A recent study examined how immigration-related stressors (e.g., fear of deportation, family separation, lack of family support) not only pose barriers to accessing prenatal healthcare, but also are emotional influences affecting PGT decisions [27]. For instance, separation from one’s nuclear family compromised the availability of family support to make PGT decisions [27]. This may explain why Learman and colleagues (2003) found that compared to other racial/ethnic groups, family influence on PGT decisions was lowest among Latina women [40]. Further, separation from older children (remaining in the country of origin) led to feelings of guilt, as these children were not provided with the same health care opportunities as children born in the U.S. [27].

## Discussion

The scoping review of the literature identifies three main themes relating to Latinx individuals’ knowledge, values, preferences and experiences around PGT: (1) genetic knowledge/literacy, (2) provider (mis)communication/patient satisfaction, and (3) cross-cultural beliefs. First, we found that even after receiving informational/educational interventions for PGT, Latinx individuals’ knowledge remained relatively low. However, when genetic information was provided by linguistically concordant providers (e.g., Spanish-speaking providers), Latinx individuals were more likely to understand and retain information about PGT. Thus, it appears that observed gaps in comprehension and knowledge may largely be attributed to language barriers. Such findings are not unique to the field of PGT. A number of public health studies have used *promotoras* or community health workers (CHWs) to relay important health information and improve comprehension, knowledge and community buy-in. A recent systematic review [50] of global studies utilizing CHWs found that CHW interventions can be highly effective for increasing credibility and buy-in from participants as well as supporting long-term sustainability of public health programs [50]. It is well-documented that Latinx individuals accept genetic testing at lower rates than their White and Black counterparts [8]. Thus, it seems that future interventions could incorporate CHWs to increase PGT knowledge and support high quality testing decisions—thereby decreasing racial/ethnic disparities. To achieve maximum benefits, the optimal timing of these interventions (prior to or during pregnancy) should also be considered [51].

The second theme identified ineffective provider-patient communication and low levels of engagement with limited interaction between Latinx individuals and their providers around PGT. The literature suggests that Latinx individuals frequently have questions related to pain accompanying testing procedures, cost of PGT, and risks of the tests for the fetus. These findings are particularly salient given the increasing role of non-invasive PGT (e.g., NIPT using cf-DNA). Our findings point to future interventions that ensure clear provider communication describing the importance of prenatal genetic counseling to Latinx individuals. Similarly, transparent presentation of risks and benefits should be provided in plain language and a culturally relevant manner. Data indicate that some Latinx individuals prefer a provider-driven approach [10]. Thus, part of culturally appropriate pre-test counseling should include eliciting decision-making preferences (e.g., provider-driven, shared, autonomous) complemented by interventions that aim to empower patients to be actively engaged in the decision-making process (e.g., promoting shared decision-making). However, it is imperative that interventions promoting shared decision making use a culturally grounded approach whenever possible (e.g., *promotoras*). Specifically, using a culturally grounded approach means that shared decision making may not just involve the patient alone. Rather, the decision-making process may involve others based on the patient’s preferences (e.g., patient’s family and/or community members) [52].

Finally, included studies indicate that Latinx individual’s religiosity, as well as immigration status, are key barriers to PGT. Culturally, religiosity plays an important role in the lives of pregnant Latinx people [53] and many people rely on prayer and other non-Western approaches (not based on Western medicine) to ensure a healthy pregnancy. Therefore, it is important that future interventions consider such religious and cultural perspectives to promote access to PGT and support high quality decisions that are informed and aligned with the values and preferences of Latinx people. Moreover, the immigration experience and immigrant status are relevant and should be considered in the context of prenatal genetic counseling for Latinx individuals. It is worthwhile to note that Latinx ethnicity and immigration status often intersect [54]. As such, it is appropriate and necessary to address client concerns that may be strictly related to immigration. For instance, it may be necessary for interventions to include content to assess and address how pregnancy is viewed in Latin American countries and particular customs and practices that surround the birth of the baby. Working with Latinx immigrants may also entail creating a space and opportunities for teleconsultations (e.g., video calls) to include family members who still reside in the country of origin. Such approaches could place additional responsibilities on already busy clinicians, yet adopting a culturally empowered approach to pre-test counseling appears crucial for surmounting disparities faced by Latinx people and may help support high-quality health decisions for PGT. Approaches that engage transnational families may improve the quality of pre-test counseling interventions and enhance PGT among Latinx people.

Overall, the results from this scoping review suggest that future interventions to promote prenatal genetic counseling among Latinx individuals must be culturally grounded. Viable approaches to promote culturally empowered care may include the use of community-based participatory approaches such as involving CHWs and other key community stakeholders (e.g., religious leaders, community organizers) to bolster engagement and improve acceptability of PGT. Further, leveraging technology (e.g., telemedicine, virtual counseling) to include family members (living in the U.S. and abroad) in decision-making is an additional way to support high quality PGT decisions among Latinx people. Third, culturally inclusive practices that consider religiosity as a cultural norm in decision-making may support more tailored approaches that are not exclusively based on Western medical practices. Currently, there is a paucity of data using such approaches for Latinx individuals in relation to PGT. Well-designed interventional studies that include cultural and community stakeholders are needed to understand how the interventions can ameliorate the identified barriers for Latinx people in relation to PGT.

With the technological advances and racial/ethnic disparities in genomic healthcare and precision medicine, this scoping review is unique and comes at an opportune time for the changing socio-political landscape. However, this review has some limitations. First, a majority of studies 20/35 (57%) were from the 1990s and 2000s. This was especially true for studies that comprised the cross-cultural beliefs theme—where 2/12 (17%) studies were from the last decade. This is problematic both because of the tremendous technological advances in gene sequencing over the past decade [2] and shifting demographics of the Latinx population in the United States. Today, fewer Latinx people are foreign-born and they come from an increasingly diverse set of Latin American countries compared to prior decades [55]. The risk of bias assessment allowed us to partially offset this limitation. Studies conducted in the 1990s and 2000s had moderate to high risk of bias. We, therefore, did not give as much weight to these studies when considering directions of future research and intervention development. Second, one team of investigators focused on this topic in the 1990s and 2000s and it is unclear if they were utilizing the same sample. Specifically, 11/35 (31%) studies were from this team of investigators and we recognize that disproportionate weighting may bias our conclusions. Third, few studies utilized valid measures to assess for genomic knowledge pointing to a need of developing culturally validated measures. It is worthwhile to note that there is a shifting sense of terminology used to describe identity. As such, the terms Latinx are more inclusive than the previous use of Latina. Similarly, pregnant women may not accurately reflect the gender identify of individuals with a uterus who do not identify as women—and thus pregnant persons would be a more inclusive term. Cumulatively, the identified limitations highlight the need for more research on knowledge, attitudes, and preferences for PGT in pregnant Latinx persons.

## Conclusion

The ‘genomic era’ has re-conceptualized our understanding of health and illness. Powerful next generation sequencing technologies and bioinformatics have enabled us to advance diagnostics and move non-invasive detection earlier and earlier (e.g., cfDNA for NIPT) [2]. However, if we are to harness the full potential of genomic discovery to improve health and wellbeing (e.g., precision medicine) of all populations alike, then we must also strive to develop and implement culturally empowered approaches to address human factors that influence decision-making for genetic testing. Culturally tailored and personalized approaches to counseling that support high quality decisions are critical to ensure acceptability of and equal access to precision medicine.

## Supplementary Information

**Additional file 1: Table S1.** JBI Critical Appraisal Checklist for Analytic Cross-Sectional Studies (response options: yes, no, unclear, n/a) included in the review. **Table S2.** JBI Critical Appraisal Checklist for Randomized Control Trials (response options: yes, no, unclear, n/a) included in the review.

## Data Availability

Not applicable.

## Abbreviations

ACOG: American College of Obstetricians and Gynecologists
AFP: Positive alpha-fetoprotein
Amnio: Amniocentesis
cfDNA: Cell-free DNA
CHW: Community health worker
CVS: Chorionic villus sampling
NIPT: Noninvasive Prenatal Testing
PGT: Prenatal genetic testing
REAL-G: Rapid Estimate of Adult Literacy in Genetics
REALM-R: Rapid Estimate of Adult Literacy in Medicine-Revised Scale
US: United States
