# Supplementary material for: Latinx individuals’ knowledge of, preferences for, and experiences with prenatal genetic testing: a scoping review
Source: Reprod Health. 2022 Jun 6;19:134. doi: 10.1186/s12978-022-01438-2 (PMC9169270; doi:10.1186/s12978-022-01438-2)
Supplement: Supplementary file 1 — Additional file 1: Table S1. JBI Critical Appraisal Checklist for Analytic Cross-Sectional Studies (response options: yes, no, unclear, n/a) included in the review. Table S2. JBI Critical Appraisal Checklist for Randomized Control Trials (response options: yes, no, unclear, n/a) included in the review. [file 12978_2022_1438_MOESM1_ESM.docx]

**Supplemental Materials**

Table A1: JBI Critical Appraisal Checklist for Analytic Cross-Sectional Studies (response options: yes, no, unclear, n/a) included in the review

|  | Browner (1996) [18] | Learman (2003) [40] | Singer (2004) [39] | Case (2007) [36] | Hawk (2011) [19] | Bryant (2015) [9] | Wagner (2018) [44] | Ault (2019) [42] | Molina (2019) [10] |
| --- | --- | --- | --- | --- | --- | --- | --- | --- | --- |
| Were the criteria for inclusion in the sample clearly defined? | No | Yes | Yes | Yes | Yes | Yes | Yes | Yes | Yes |
| Were the study subjects and the setting described in detail? | No | No | Yes | Yes | No | Yes | Yes | Yes | Yes |
| Was the exposure measured in a valid and reliable way? | Yes | Yes | yes | Yes | Yes | Yes | Yes | Yes | Yes |
| Were objective, standard criteria used for measurement of the condition? | Unclear | No | Unclear | Unclear | Yes | Yes | Yes | Yes | Yes |
| Were confounding factors identified? | No | Yes | Yes | Yes | No | Yes | Yes | Yes | Yes |
| Were strategies to deal with confounding factors stated? | No | Yes | Yes | Yes | No | Yes | Yes | Yes | Yes |
| Were the outcomes measured in a valid and reliable way? | Unclear | No | Yes | Unclear | Yes | Yes | Yes | Yes | Yes |
| Was appropriate statistical analysis used? | Yes | Yes | Yes | Yes | Yes | Yes | Yes | Yes | Yes |

Table A2: JBI Critical Appraisal Checklist for Randomized Control Trials (response options: yes, no, unclear, n/a) included in the review

|  | Kupperman (2014) [30] |
| --- | --- |
| Was true randomization used for assignment of participants to treatment groups? | Yes |
| Was allocation to treatment groups concealed? | Yes |
| Were treatment groups similar at the baseline? | Unclear |
| Were participants blind to treatment assignment? | Yes |
| Were those delivering treatment blind to treatment assignment? | Yes |
| Were outcomes assessors blind to treatment assignment? | Yes |
| Were treatment groups treated identically other than the intervention of interest? | Yes |
| Was follow up complete and if not, were differences between groups in terms of their follow up adequately described and analyzed? | Yes |
| Were participants analyzed in the groups to which they were randomized? | Yes |
| Were outcomes measured in the same way for treatment groups? | Yes |
| Were outcomes measured in a reliable way? | Yes |
| Was appropriate statistical analysis used? | Yes |
